# Phthisis and Superstition among the Moaris

**Published:** 1907-02

**Authors:** 


### PHTHISIS AND SUPERSTITION AMONG THE MAORIS.

---

Dr. Edwin Chill, Ealing, England, in the British Medical Journal for August 18, relates some interesting observations made on a visit to New Zealand. Though physically a powerful race, the Maoris are slowly dying out, like many other native populations when brought into contact with European civilization.

Of old they were divided into a number of tribes perpetually at war with one another. Their villages are built and stockaded on hills as a protection against sudden raids and they only descend to the plains to cultivate the land during times of temporary peace. Their clothing, which consisted of woolen matting, was well suited to protect their bodies from cold and damp. Since the establishment of a settled government, the Maoris have abandoned the more salubrious hills and have built their stocades on low-lying damp land. They have partially adopted European clothing, and men and women may be seen lazily standing outside their poorly-constructed huts or roaming about the streets clad in insufficient garments. This altered condition of habitation and dress, no doubt, produces in them the soil necessary for the growth and multiplication of the tubercle bacilli.

Dr. Chill states that they have also adopted European education and religion, for in every Maori village is the schoolmaster, who combines in his person the duties of lawyer, doctor, pastor, schoolmaster and friend. There is always the church, for the Maoris are all Christians now, but close by is the native meeting house with its grotesque carvings and symbols of old canabalistic days. After a visit to the wonders of Geyserland, Dr. Chill's party arrived at a village beautifully situated on the shores of Lake Taupo. There had been nine deaths the previous week among the small community of natives, and three others were then lying seriously ill. Dr. Chill was asked to see them as the nearest medical man lived some sixty miles away. Accordingly, he was conducted to the first case, an emaciated young woman of 25, with all the symptoms of acute phthisis. There was a large cavity in the left lung, and a patch of consolidation at the apex of the right. She was lying on a mat stretched on the ground in a tent some 8 by 6 feet.

The next case was that of a child who had had three attacks of hemoptysis, and, fearing a fatal termination, the parents had called in a native medicine man, who had employed charms and incantations, and then prophesied the recovery of the patient. Hearing voices as Dr. Chill and a friend approached, the mother inside the sick tent promptly extinguished the candle; however, with a little persuation, she came out and explained that she was afraid of the presence of an English medical man, as it might break the spells of the native doctor, and her child might die. They left, but not before Dr. Chill heard the patient give the characteristic cough associated with the last stage of phthisis. He was curious to know what excuse the medicine man would make for the failure of his prophecy. The reply usually made is simple enough: "If it had been a Maori complaint, recovery would have been certain, but what could he do against a pakeha (European) disease?"

The third patient lived in an outlying settlement, to which there was no road. The schoolmaster acted as guide, and conducted Dr. Chill through bush and swamp till they reached the place, about a mile and a half away. On the way the schoolmaster gave an account of his work and of his difficulties in treating patients with the aid of an elementary medical book and a case of drugs supplied by the government, as well as an account of the Maori character in health and disease.

It is well known, Dr. Chill states, that when a Maori is taken ill, as observed in some other primitive races, he will make up his mind that he is going to die and dies. Accordingly, on the first symptoms of illness, the tent is quickly fitted up in the garden adjoining, and the patient hurried into it, for should he expire in the house that house would be "tabu," after which no native would live in it. Thus it is that in various parts of the country deserted houses are seen. Superstition dies hard among primitive peoples.

This Maori settlement is situated among a number of hot springs strongly impregnated with sulphur. The whole village is enveloped in the rising steam. Some of the pools, formed by the springs, are used for bathing, some for washing clothes, and those of intense heat for boiling in kettles and cooking food. The question arises whether the constant inhabitation of this seam, boh here in other parts of Geyserland, acts injuriously on the pulmonary tissues and is the exciting cause of tuberculosis.

The Maoris now number about 40,000, compared with a population of nearly 900,000 colonists. In the South Island they are practically extinct. They are mentally and physically superior to all the other native inhabitants of Oceania and add considerably to the charm and interest of New Zealand as a tourist center. Surely they are a race worth preserving. A little outlay should procure them qualified and more accessible medical advice and sanitorium for the treatment of tuberculosis in place of their miserable tents. The great stumbling block, one can conceive, is the inborn native superstition, but even this is not an insuperable difficulty.—*Jour. A. M. A , Jan.* 12, '07.

